# Neuroinflammation and Oxidative Stress in Parkinson’s Disease, Alzheimer’s Disease, and COVID-19: Microglia–Neutrophil Interaction

**DOI:** 10.1021/acsomega.5c10397

**Published:** 2026-01-26

**Authors:** Ana B. de Araújo, Francisco V. C. S. Azul, Yandra Cardoso Carneiro, Caren N. S. de Sousa, Silvânia M. M. de Vasconcelos, Francisco J. Rios, Luzia K. A. M. Leal

**Affiliations:** † Center of Cosmetics and Pharmaceutical Studies, Department of Pharmacy, Federal University of Ceará, CEFAC, Fortaleza, CE 60430-372,Brazil; ‡ Neuropharmacology Laboratory, Department of Physiology and Pharmacology, Federal University of Ceará, NPDM, Fortaleza, CE 60430-270,Brazil; § Research Institute of the McGill University Health Centre, Montreal, QC H3H 2R9,Canada

## Abstract

Abnormal activation of the immune system and oxidative stress are crucial factors in neurodegenerative disorders, such as Parkinson’s disease and Alzheimer’s disease. Microglia, neutrophils, oxidative stress mediators such as reactive oxygen species (ROS), lipid peroxidation products (e.g., malondialdehyde), and nitrosative stress markers (e.g., nitrite and nitrate) play important roles in neuroinflammatory mechanisms. Microglial cells acquire a proinflammatory phenotype through interactions with endogenous or exogenous compounds, including cell debris, abnormally modified proteins (including Aβ species and alpha-synuclein), and pathogens (e.g., SARS-CoV-2). They produce many inflammatory mediators and promote the activation of adjacent brain cells and leukocyte infiltration, including polymorphonuclear neutrophils. Accumulation of neutrophils in the central nervous system (CNS) leads to the secretion of more proinflammatory mediators, such as cytokines, proteases, and oxidants, and the formation of neutrophil extracellular traps (NETs). These processes are associated with the pathological activation of microglial cells, cell death, consequent influence on neuronal functions, or even neuronal death, which is a hallmark of CNS disorders. In this review, we address the importance of inflammatory mechanisms and oxidative stress in the CNS associated with Parkinson’s disease, Alzheimer’s disease, and the neuronal effects observed in coronavirus disease 2019 (COVID-19), as observed by the abnormal activation of central and peripheral immune cells, such as microglia and neutrophils. We also discuss emerging evidence linking SARS-CoV-2 infection to neuroinflammatory mechanisms that could contribute to neurodegenerative complications.

## Graphical abstract

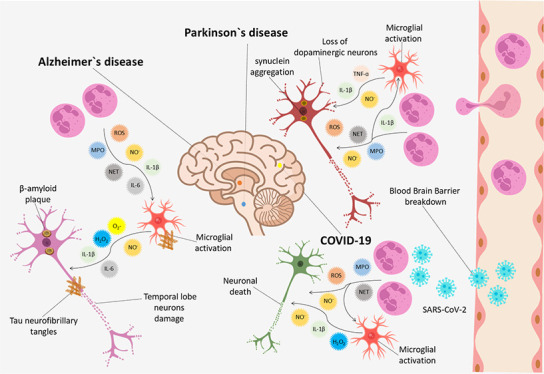

## 1 Neuroinflammation in Classical Neurodegenerative Diseases and COVID-19

Inflammation and oxidative stress are closely interconnected across a spectrum of infectious and noninfectious disorders, including Parkinson’s and Alzheimer’s diseases, and coronavirus disease 2019 (COVID-19), which affect both peripheral organs (heart, kidneys, and liver) and the central nervous system (CNS).^1,2^ These conditions share several clinical manifestations, most notably cognitive decline and impairments in attention and concentration,^3^ which are linked to neuronal dysfunction and loss. Within this framework, it is important to emphasize the role the CNS-resident immune system, in coordination with the peripheral immune system, which interact dynamically from disease onset to its exacerbation.^4^

Although knowledge of the consequences of the COVID-19 infection is still in progress, some studies indicate that it might be significant to Parkinson’s disease and Alzheimer’s.^5^ Recent clinical case reports suggest that severe acute respiratory syndrome coronavirus-2 (SARS-CoV-2) could be responsible for initiating the demyelination process.^6^ Additionally, biopsies from deceased patients revealed endothelial vasculopathies, microhemorrhages, and microthrombi in brain tissues, indicating the harmful effects of the infection on the CNS.^4^

Thus, this study starts from the hypothesis that Alzheimer’s Disease, Parkinson’s Disease, and COVID-19 share convergent pathogenic pathways mediated by neuroinflammation and oxidative stress, suggesting that the processes triggered by viral infection may act as triggers or accelerators of neurodegenerative mechanisms already described in chronic diseases of the central nervous system. The aim of this work is, therefore, to integrate recent evidence supporting this mechanistic interconnection, discussing how systemic inflammatory responses and redox imbalance promoted by COVID-19 may contribute to neuronal vulnerability and the progression of neurodegenerative disorders.

## 2 Oxidative Stress and Neuronal Damage

Oxidants play important functions in homeostasis, including cell signaling,^7^ immune response,^8^ smooth muscle relaxation,^9^ blood pressure modulation,^10^ and neuronal development.^11^ Free radicals and nonradicals are produced in subcellular organelles and structural components, including the plasma membrane (lipid bilayer), mitochondrion, cytoplasm, peroxisome, and endoplasmic reticulum.^12^ Oxygen, nitrogen, and sulfur are the main elements for oxidant formation, such as reactive oxygen species (ROS), reactive nitrogen species (RNS), and reactive sulfur species (RSS), respectively.^13^ Main free radicals include superoxide anion (O_2_^.‑^), hydroxyl radical (OH·), and nitric oxide (NO), whereas nonradicals comprising hydrogen peroxide (H_2_O_2_), hypochlorous acid (HOCl), and peroxynitrite (ONOO^–^) are derived from NO by reacting with O_2_^.‑^. RSS are formed by thiol reduction, resulting in sulfonic acid production.^14^ Furthermore, interactions between cellular sources of free radicals and nonradicals are significant: for instance, the O_2_^.‑^ released in the mitochondrial matrix can damage DNA, while its release into the intermembrane space contributes to its elevation in the cytosol.^12,15^

Among the complexes of the mitochondrial respiratory chain, Complex I (NADH: ubiquinone oxidoreductase) and Complex III (ubiquinone: cytochrome c oxidoreductase) are significant sources of O_2_^.‑^, H_2_O_2_, and the OH·. The oxidants production by Complex I is mediated by the reduced flavin, which reacts with O_2_ to generate O_2_^.‑^, which rapidly dismutates into H_2_O_2_, while OH· is produced from the interaction of O_2_^.‑^ or H_2_O_2_ with metal ions such as iron or copper. The production of oxidants by Complex III is mediated by electron transfer through iron–sulfur centers and interaction with reduced ubiquinone, generating O_2_^.‑^ that can also be converted into H_2_O_2_ and subsequently lead to the formation of OH·. Mutations or dysfunctions in Complex I or Complex III are associated with progressive cellular damage, especially in neurons, and plays an important role in the pathogenesis of neurodegenerative diseases, such as Parkinson’s and Alzheimer’s diseases.^16,17^

In the human body, ROS, RNS, and RSS exert dual effects, displaying both harmful and beneficial properties.^13,18^ Oxidative stress occurs when the balance between oxidant production and antioxidant defense is disrupted in favor of oxidants.^19,20^ Antioxidant mechanisms include both enzymatic and nonenzymatic systems. The nonenzymatic system includes antioxidants such as ascorbic acid and lipoic acid, polyphenols, and carotenoids derived from dietary sources. Enzymatic antioxidants include superoxide dismutase (SOD), catalase (CAT), glutathione peroxidase/reductase (GPx/GR), and peroxiredoxin,^21^ which react with high or low molecular weight molecules, such as γ-l-glutamyl-l-cysteinyl-glycine [also known as glutathione (GSH)], pyruvate, amino acids, transferrin, ferritin, and ceruloplasmin. However, when these antioxidant systems become insufficient, oxidative stress products accumulate in the body and contribute to the development and progression of several inflammatory disorders, as it has been observed in Parkinson’s disease, Alzheimer’s disease, amyotrophic lateral sclerosis, multiple sclerosis, hereditary spastic paraplegia, and COVID-19 ^22,23^ (Figure 1).

**Figure 1:**
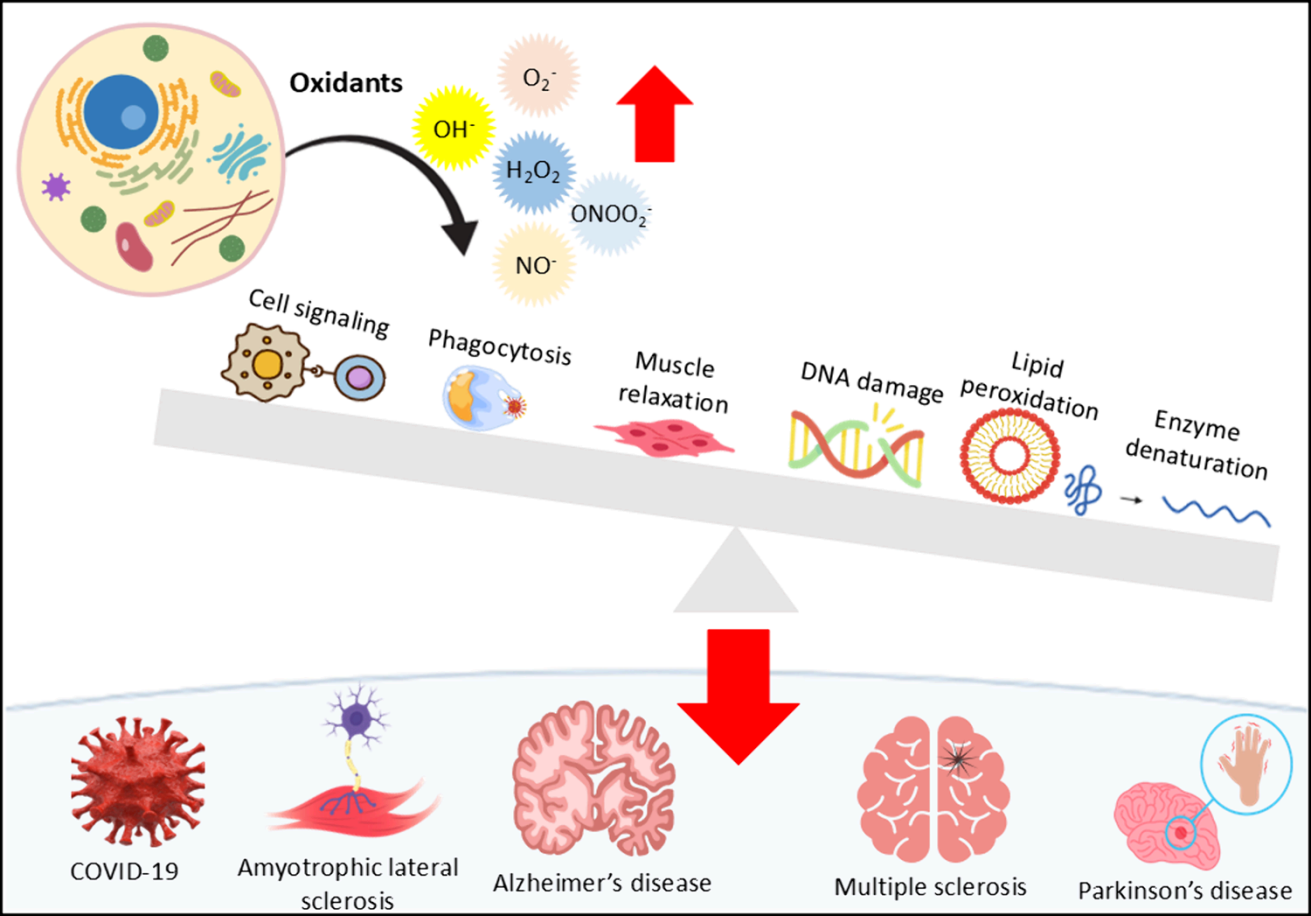
In physiological conditions, oxidants are released by cell organelles and play important functions in cell signaling, phagocytosis, and muscle relaxation. However, deleterious effects occur under oxidative stress, when the balance between oxidant generation and the antioxidant system is affected, leading to lipid peroxidation, enzymatic denaturation, and DNA damage. Oxidative damage mediators such as ROS, lipid peroxidation byproducts, and nitrosative stress markers accumulate in the organism and contribute to the development and progression of several neuroinflammatory diseases, such as Parkinson’s disease, Alzheimer’s, amyotrophic lateral sclerosis, multiple sclerosis, and COVID-19.

The autoxidation of soluble cellular components, such as hydroquinones, flavins, thiols, hemoproteins, and catecholamines, generates superoxide radical, which is an important precursor of other oxidants. Aldehyde oxidase, flavin dehydrogenases, and dihydroorotic acid dehydrogenase are enzymes involved in this process, especially the flavoenzyme xanthine oxidase (XOR).^24^ Xanthine dehydrogenase (XDH, NAD+-dependent form) and xanthine oxidase (XOD) are separate enzymes; however, they exhibit exchangeable forms. Under physiological conditions, XDH shows high activity, utilizing hypoxanthine or xanthine as substrates and NAD as a cofactor to produce NADH and uric acid. XOD competes for the same substrates as XDH but uses O_2_ as a cofactor to O_2_^.‑^ and uric acid. However, under oxidative stress, there is an imbalance of XDH/XOD, favoring XOD through irreversible proteolysis or oxidation of the thiol groups in the cysteine structure. Conversion of XDH to XOD is also dependent on sulfhydryl oxidants by reversible reactions in similar proteolytic cleavage sites.^25^

The oxidant functions of uric acid are still contradictory. It has been described that uric acid plays an important role as an antioxidant agent by preventing modifications in lipids and proteins, contributing to the protection of neural cells in brain development.^26,27^ The uric acid antioxidant activity seems to be related to properties involving iron chelation and scavenging of free radicals, such as OH·, singlet oxygen, hypochlorous acid, oxoheme oxidants, hydroperoxyl radical, and ONOO^–^. Clinical studies have suggested that serum uric acid levels are typically lower in patients with idiopathic neurodegenerative diseases than in healthy controls. This finding may, at least in part, account for the increased oxidative stress observed in patients with Parkinson’s disease or Alzheimer’s.^28,29^ Elevated serum uric acid levels, however, have also been associated with several health disorders, including gout, renal dysfunction, and cardiovascular diseases. Nevertheless, some reports indicate that the antioxidant capacity of uric acid is relatively weak and that it may serve as a biomarker of cell death.^30^ Therefore, the physiological significance of uric acid appears to vary across organs and warrants further investigation.

Superoxide anion is converted by SOD to produce H_2_O_2_, a nonradical species that is relatively stable, but in the presence of metals (e.g., Fe^2+^ or Cu^+^), it is converted into OH· (Fenton reaction). This radical is highly reactive, and there is no known enzymatic defense to promote cellular protection against its deleterious effects.^31^ Hydroxyl radical has low specificity and acts mainly on thiol groups of proteins, inducing changes in structure and/or function. These deleterious effects are partially reduced by thioredoxin and peroxiredoxin.^32^

There is an important interaction between reactive species produced during oxidative stress, which can interfere with each other’s formation or act in a synergistic manner in biological systems. NO is generated by a family of specific NO synthases (NOS) expressed by various cell types and has an important function in brain cell communication. However, in the presence of O_2_^.‑^, other RNS are formed, including neurotoxic ONOO^–,^ nitrogen dioxide (NO_2_), dinitrogen trioxide (N_2_O_3_), and dinitrogen tetroxide (N_2_O_4_).^33^ At high concentrations, ONOO^–^ in the CNS induces lipid peroxidation, which affects neuronal Ca^2+^ homeostasis. High levels of ONOO^–^ have been detected in Alzheimer’s disease and are associated with the expression of a dysfunctional form of NOS, known as uncoupled NOS, which produces O_2_^.‑^ instead of NO and inhibit tyrosine phosphorylation. In addition, uncoupled NOS suppresses the receptor for nerve growth factor (NGF), thereby impairing signaling and neural function, and contributes to oxidative stress and, consequently, neurodegenerative processes.^34^

Immune cells, including macrophages, neutrophils, and lymphocytes, actively participate in neuroinflammatory disorders by producing ROS, RNS, and inflammatory cytokines, which have effects on acute and chronic inflammation. Technological advances in diverse fields, including biochemical, genetic, imaging, and artificial intelligence, have allowed a better understanding of how the neuroinflammatory response contribute to the development of neurological disorders induced by various factors, including viral infections such as SARS-CoV-2. A deeper insight into molecular pathways may aid in identifying potential pharmacological targets.^35^

## 3 Immune Cell Dynamics in CNS Disorders: Microglia and Neutrophils

Microglial cells and neutrophils have distinct embryonic origins. Microglia are derived from yolk sac progenitors during early embryonic development, migrating to the CNS in the first trimester of gestation. This origin makes microglia unique among resident immune cells in the brain, with a specialized role in maintaining homeostasis and responding to injuries in the CNS.^33,36^ In contrast, neutrophils originate from hematopoietic stem cells in the bone marrow and are part of the myeloid lineage. They are recruited to the CNS during infections or inflammatory processes in response to signals such as cytokines.^37^

Inflammation in the CNS is orchestrated by resident immune cells, such as microglia, as well as by infiltrating peripheral immune cells, including neutrophils. Microglia, the primary resident immune cells of the CNS, are essential for maintaining homeostasis and responding to injury or infection. They are highly plastic and can adopt either pro-inflammatory or anti-inflammatory phenotypes, thereby both responding to and shaping the surrounding cellular environment. In neurodegenerative diseases, interactions between microglia and astrocytes contribute to the amplification of inflammatory responses. In addition, disruption of the blood-brain barrier (BBB) facilitates the infiltration of peripheral immune cells, including mononuclear and polymorphonuclear cells such as neutrophils. Once activated, these cells exert antimicrobial functions through the production of ROS, RNS and HOCl acid, degranulation with the release of chemical mediators (e.g., cytokines and proteases), as well as the formation of neutrophil extracellular traps (NETs), all of which further amplify CNS inflammation.^38,39^

The brain has physiological characteristics that increase its vulnerability to the harmful effects of oxidative stress, such as (i) it consumes approximately 20% of the total oxygen (metabolizes up to ∼ 4 × 10 ^21^ of glucose/min); (ii) it is an environment rich in lipids**;** (iii) high production of oxidants (ROS, RNS and RSS) by neurochemical reactions; and (iv) increased deposition of metals (e.g., Fe^2+^ and Cu^2+^) in aging process, which favors the production of additional free radicals, such as OH· by Fenton reaction.^40,41^

Neuroinflammatory response plays a crucial role in the development of neurological diseases. Inappropriate activation of immune cells in the CNS leads to production of soluble mediators that can transiently or permanently affect neuronal function or induce neuronal death, as observed in neurological disorders and viral infections. Oxidative stress mediators and cytokines are among the main mediators associated with the development of these diseases.

In CNS, major producers of ROS, RNS and cytokines are astrocytes and phagocytic cells, such as microglia (resident cells) and infiltrated leukocytes with elevated expression of nicotinamide adenine dinucleotide phosphate oxidase (NADPH oxidase), inducible nitric oxide synthase (iNOS) and XOD.^8^ Glial cells, also called neuroglia, are divided into two large groups, with distinct functions and morphology: macroglia and microglia. Macroglia comprises different cell populations with distinct structures and functions, including oligodendrocytes, astrocytes, Schwann cells, and ependymal cells.^42^ Astrocytes are the most common glial cells in the brain and exhibit multifunctional properties. They regulate blood flow, maintain the BBB, supply energy metabolites to neurons, modulate synaptic activity, control neurotrophin secretion, remove dead cells, and regulate the extracellular ionic balance.^43,44^

### 3.1 Microglia

These cells were first described by Pío del Río Hortega, who demonstrated the morphology and phagocytic functions of these cells in 1932.^45^ They are the only innate immune cells in the CNS and represent 0.5% to 16.6% of the total cell population in the human brain. This variation is related to the anatomical region or activation phenotype.^46,47^

Microglia, along with other CNS cells such as astrocytes, oligodendrocytes, and migrating leukocytes, are considered sentinel cells responsible for tissue integrity. Traditionally, microglial cells are considered “CNS macrophages,” not only because they show similar activity in tissues but also because they exhibit common markers, such as CD11b and CD68. Previous studies have demonstrated that microglia are highly dynamic in brain homeostasis. Microglial cells modulate neuronal activity, and in return, neurons may also interfere with microglial functions. The functional effects of microglia depend on cell–cell contact or on the production of soluble factors, including neurotransmitters and the chemokine CX3CL1/fractalkine. Moreover, the receptor for this chemokine (CX3CR1) is upregulated in microglia and neurons in pathological conditions.^48^ These bidirectional communications seem to be essential for the maintenance of brain homeostasis. Furthermore, microglia in aged brains exhibit morphological changes that affect their homeostatic functions.^48,49^

In the CNS, microglia and astrocytes interact with damage-associated molecular patterns (DAMPs) (cell debris, modified proteins, such as amyloid beta protein (Aβ) species and α-synuclein, and oxidized lipids) or pathogen-associated molecular patterns (PAMPs) (fragments of bacteria or viruses, including SARS-CoV-2), through pattern recognition receptors (PRRs) such as Toll-like receptors (TLRs) and nucleotide-binding oligomerization domain-like receptors (NLRs). After interacting with PAMPs and/or DAMPs, PRRs located on the cell surface or in the intracellular environment activate intracellular signaling pathways, including adaptor molecules, kinases, and transcription factors such as mitogen-activated kinases (MAPK), Janus kinase-signal transducer and activator of transcription (JAK-STAT), nuclear factor kappa B (NF-κB), and activator protein 1 (AP-1). These activated cascades induce the production of a broad spectrum of mediators, including cytokines (IL-6, TNF-α, IL-1β), chemokines (MCP-1, IL-8), and adhesion molecules, which collectively orchestrate the activation of the innate immune response and serve as a crucial link to the activation of the adaptive immune response.^34^

Once activated, microglial cells acquire either the M1 phenotype (proinflammatory) or M2 (anti-inflammatory) phenotype. In this context, it is important to emphasize that microglial reactivity represents a dynamic and continuous phenotypic process, which partly explains why this binary classification is limited in vivo. In their nonactivated state, microglia cells have a small cell body with long, thin, and ramified branches that extends into surrounding tissue. However, microglial morphology is highly plastic, and when activated, they undergo significant morphological and functional changes according to the type and severity of the stimulus. The M1 phenotype cells have amoeboid form with their branches retracted and enlarged body.^50^ This morphology is characterized by increased phagocytosis and production of pro-inflammatory chemokines and cytokines, including interleukin 1β (IL-1β), tumor necrosis factor alpha (TNF-α), and IL-6, as well as an increased expression of surface markers such as CD16/32, CD86, CD40, and inducible NO synthase, contributing to neuroinflammation response.^33,34^

The M2 phenotype (alternative) is associated with efferocytosis (phagocytosis of apoptotic cells) and the release of anti-inflammatory mediators such as IL-10, transforming growth factor-β (TGF-β), and brain-derived neurotrophic factor (BDNF).^51^ Morphologically, M2 microglia exhibit a more ramified, branching appearance, reflecting their role in tissue repair and homeostasis. This phenotype promotes neuroprotection and tissue repair tby facilitating the clearance of dead cells and the secretion of neurotrophic factors. Functionally, M2 microglia support neuronal survival and regeneration by promoting anti-inflammatory responses and reducing damage caused by excessive inflammation. Additionally, M2 microglia play a critical role in tissue repair and remodeling in the CNS, particularly after injury.^50^

### 3.2 Neutrophils

Neutrophils play crucial roles in the innate immune response against microorganisms, primarily through the production of ROS, lipid mediators, granule release, and formation of NETs. Current knowledge shows that they are relatively short-lived, surviving only for a few hours in circulation before undergoing apoptosis. Hence, they are constantly formed by the bone marrow.^52,53^ However, it has also been demonstrated that they might survive up to 5.4 days under homeostatic conditions,^54^ an effect that can be augmented in a pro-inflammatory microenvironment, especially in the presence of IL-17, IL-8, interferon-γ (IFN-γ), TNF-α, and granulocyte–macrophage colony-stimulating factor (GM-CSF).^55,56^

Activation of neutrophils during infection, tissue damage, or endogenous insults triggers various cell signaling systems crucial to the innate and adaptive immune response. These processes involve PRRs (e.g., Toll-like receptor (TLR) family and C-type lectin receptor dectin-1), kinases, and transcription factors. After activation, neutrophils display a wide range of responses associated with degranulation, phagocytosis, and formation of NETs, producing a massive repertoire of chemical mediators including oxidants, granule proteins, cytokines, and lipid mediators.^57^

Additionally, neutrophils produce neutrophil-derived pattern recognition molecules (PRMs), including collectins, ficolins, and pentraxins, which possess antibody-like properties by promoting opsonization and activation of the complement system. Therefore, neutrophils play a critical role in maintaining or restoring homeostasis. Neutrophil death may occur through apoptosis, necrosis, or formation of NETs, and the resolution of the inflammatory process is characterized by the removal of dead neutrophils by macrophages in a process called efferocytosis.^58^

The formation of NETs was first described in 2004 by Brinkmann et al.^59^ in neutrophils activated with phorbol myristate acetate. It is considered a cell death program distinct from apoptosis or necrosis, and dependent on plasma membrane lysis. Furthermore, in vivo studies have demonstrated NET formation and the trapping of *E. coli* in hepatic sinusoids by intravital microscopy.^60^ Dynamically, NETs are formed by activated neutrophils that release web-like structures of DNA coated with histones, granule proteins (e.g., MPO and elastase), and cathepsin G. In addition, NETosis depends on the generation of reactive oxidants by NADPH oxidase. Studies have shown that NETs are also formed in response to inflammatory stimuli such as microorganisms and their released products (lipopolysaccharides, cytokines, chemokines, and lipid mediators), with each having distinct mechanisms and contents. Moreover, NETs formed and released from mitochondrial material exhibit nonlytic properties and do not contain histones in their structure compared to those formed in the nucleus.^61,62^

It has been demonstrated that neutrophils play an important role in the pathogenesis of various neuroinflammatory disorders, Parkinson’s and Alzheimer’s disease.^8,63,64^ Neutrophils have limited access to the brain parenchyma due to the BBB. However, during infection, trauma, or neurodegeneration, they rapidly infiltrate the CNS to provide protective functions. This defensive role, however, can become deleterious in the setting of chronic neuroinflammation, where excessive NET release leads to tissue damage and contributes to disease progression.^65^ These happen when neutrophils lose physiological functions and assume a pathogenic phenotype characterized by abnormal cell activation. Although increasing evidence indicates that neutrophil accumulation and NET formation contribute to neuroinflammation, the exact role of these mechanisms in the initiation of neurodegenerative remains unclear.^66^ Recent studies have shown neutrophil infiltration and NET formation in the brains of patients and animal models of Alzheimer’s disease, linking these mechanisms to cognitive decline and vascular dysfunction.^67,68^ Thus, although neutrophil activity is essential for host defense, their excessive and persistent activation in the CNS may exacerbate neural damage, highlighting the need for further investigation into their casual role.

In light of this, therapeutic strategies aimed at reducing the formation of NETs have shown promise, including pharmacological inhibition of peptidylarginine deiminase 4 (PAD4), extracellular DNA degradation with DNase I, and innovative targeted delivery approaches, such as DNase I-mediated chemotactic nanoparticles, which accumulate in regions of NET formation, effectively degrade these structures, and remodel the inflammatory microenvironment.^71,72^

In experimental models of diseases that affect the CNS, such as Alzheimer’s disease and Parkinson’s disease, NETs are associated with BBB disruption and microglia activation through PRRs, such as TLR2 and TLR4, amplifying the production of proinflammatory mediators, such as IL-1β, TNF-α, type I interferon, and NO. This reciprocal interaction between NETs and microglia establishes a vicious cycle of inflammation and oxidative imbalance that accelerates neuronal loss.^66^ Thus, It is worth noting that recent studies^66,69,70^ have demonstrated that the structural components of NETs, such as citrullinated H3 histones, MPO, matrix metalloproteinases (MMPs), and neutrophilic elastase, exert direct neurotoxic effects on neurons by promoting oxidative stress, mitochondrial dysfunction, and neuronal apoptosis. In light of this, therapeutic strategies aimed at reducing the formation of NETs have shown promise, including pharmacological inhibition of peptidylarginine deiminase 4 (PAD4), extracellular DNA degradation with DNase I, and innovative targeted delivery approaches, such as DNase I-mediated chemotactic nanoparticles, which accumulate in regions of NET formation, effectively degrade these structures, and remodel the inflammatory microenvironment.^71,72^

In COVID-19, exacerbated infiltration and activation of leukocytes/neutrophils are associated with poor clinical status. Therefore, restoration of normal neutrophil function is required, and attenuation of NETs formation might represent a therapeutic strategy.^73^

Under physiological conditions, the brain is considered an immune-privileged region, and the BBB plays an important role in limiting the access of peripheral immune system cells to brain tissues. However, under inflammatory conditions there is an increase in leukocyte migration (e.g., neutrophils), from postcapillary venules to the brain by crossing the BBB. During these inflammatory processes, activated microglia release proinflammatory cytokines and chemokines, such as IL-1β and TNF-α, which can compromise the integrity of the BBB. These mediators activate endothelial cells within the BBB, increasing its permeability and allowing the infiltration of neutrophils from the bloodstream into the brain parenchyma. This cascade of events not only enables neutrophil migration but also amplifies the inflammatory microenvironment in the CNS.^74^

Neutrophils were previously considered transcriptionally inactive cells. However, advanced techniques such as single-cell transcriptomics have revealed that neutrophils are capable of substantial gene expression and release various inflammatory mediators.^55,75,76^ These mechanisms are potentiated by the participation of other cells from the immune and vascular systems, including activated astrocytes, endothelial cells, lymphocytes, and macrophages^55,77^ Hence, accumulation of neutrophils in the CNS leads to secretion of more proinflammatory factors, which in turn increases the activation of microglial cells and consequent neuronal damage (Figure 2).

**Figure 2:**
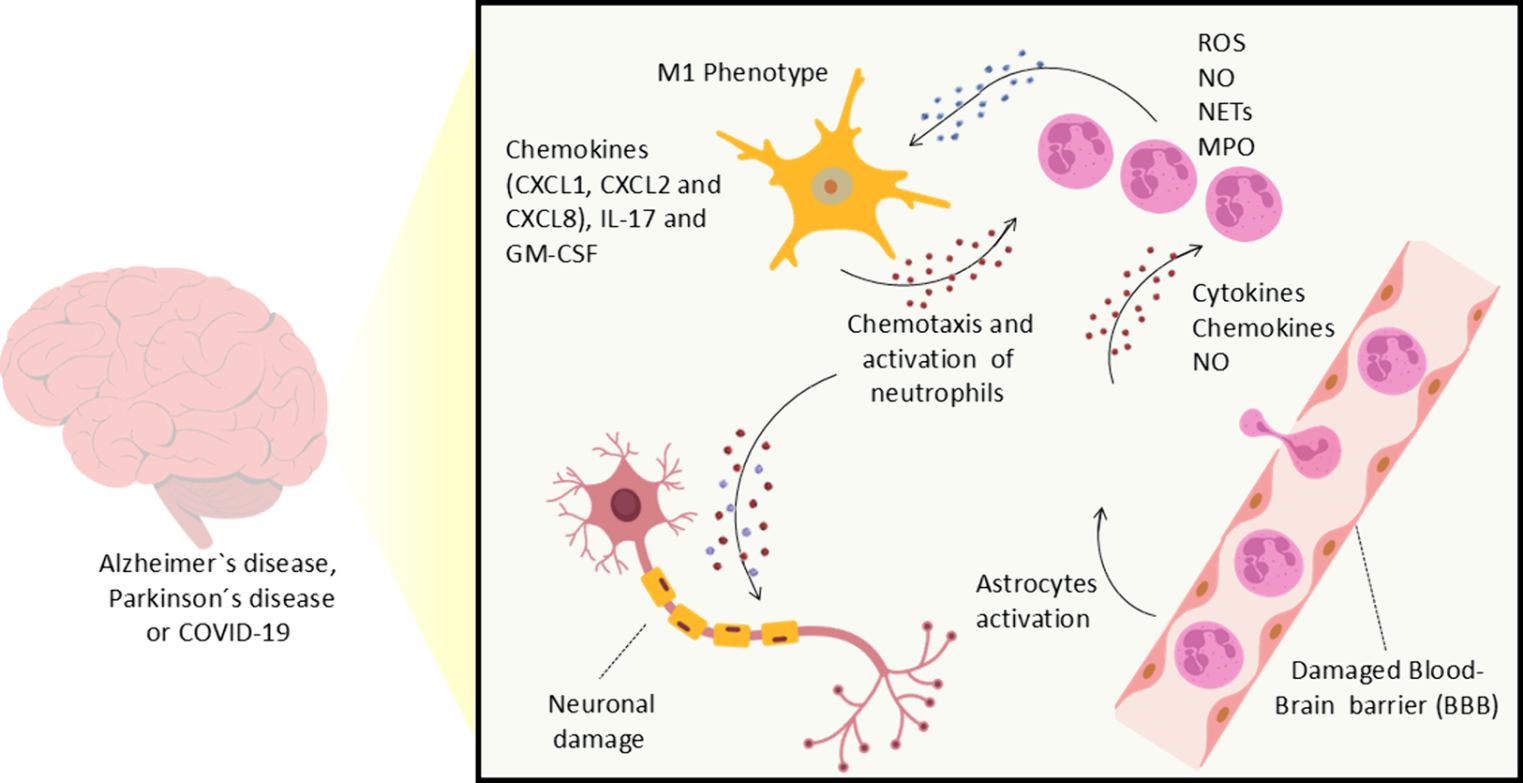
Crosstalk between microglial and neutrophils. Brain injury and neurodegeneration associated with loss of redox potential lead to astrocyte activation and increased permeability of the BBB, resulting in leukocyte infiltration that amplifies BBB damage. In the brain, neutrophils may interact with other cells, such as microglia. Activated microglia (M1-proinflammatory phenotype) release various cytokines, chemokines, and growth factors, including TNF-α, IL-1β, IL-12, GM-CSF, CXCL1, CXCL2, CXCL8, and GM-CSF. This inflammatory microenvironment contributes to enhanced chemotaxis and neutrophil activation. Activated cells produce ROS, RNS, release their granule contents (e.g., myeloperoxidase) and promote the formation of NETs. Microglial-neutrophil interactions act on neurons, inducing a loss of neuronal function (permanent or transient) or death, hallmark features of neurodegenerative diseases and neuroinflammation associated with viral infections, including COVID-19. Additionally, similar mechanisms of redox potential loss may be involved in external injuries, suggesting a common role in neurodegeneration.

Park et al.,^78^ using human cells in an interesting microfluidic system mimicking central-peripheral innate immunity in Alzheimer’s disease, observed that β-amyloid-activated human microglia cells produced chemoattractant mediators for neutrophils (e.g., IL-8, CCL2, CCL3/4, and CCL5), which in turn released other inflammatory mediators, including IL-2 and macrophage inhibitor factor (MIF). Interleukin 2 (IL-2) and MIF are known to amplify the proinflammatory mechanisms of human neutrophils and upregulate neuroinflammation in Alzheimer’s disease. Blood-derived leukocyte subpopulations, including neutrophils, have been identified in the brains of patients with Parkinson’s and Alzheimer’s disease and in corresponding animal models.^79,80^ Moreover, neutrophils seem to indirectly modulate neurodegeneration and communicate with other immune cells. As a matter of fact, neutrophil depletion or leukocyte function-associated antigen-1 (LFA-1) integrin inhibition improved memory and cognitive function in mice in experimental Alzheimer’s disease.^81^

Microglial activation and neutrophil infiltration play central roles in the progression of neuronal injury. These processes are orchestrated by molecular mechanisms that involve the activation of PRRs, such as TLRs and NLRs, which trigger key intracellular signaling pathways, including NF-κB and MAPK (ERK, JNK, and p38), that modulate the immune response in the CNS. The NF-κB pathway, in particular, acts as a central point of convergence between oxidative stress and inflammation by promoting the transcription of proinflammatory genes such as proinflammatory cytokines (IL-1β, IL-6, TNF-α), iNOS, and COX-2, thereby perpetuates microglial activation and tissue damage. Similarly, the MAPKs cascade (ERK1/2, JNK, and P38) contributes to the phosphorylation of transcription factors and effector proteins that amplify the inflammatory response and oxidative stress, resulting in mitochondrial dysfunction, ROS production, and neuronal death.^82,83^

Among the mechanisms recently described, the activation of the NOD-like receptor pyrin domain containing 3 (NLRP3) inflammasome, an intracellular multiprotein complex that activates the production of the cytokines IL-1β and IL-18, exacerbating neuroinflammation and contributing to the worsening of CNS diseases, such as Parkinson’s disease, Alzheimer’s disease, and MS, stands out. This research demonstrates that oxidative stress, mitochondrial dysfunction, and the release of DAMPs (such as ATP, α-synuclein crystals, and oxidized protein fragments) are strong inducers of NLRP3 activation in microglia. The persistence of this activation contributes to the maintenance of a neurotoxic environment, with increased BBB permeability and recruitment of peripheral leukocytes, such as neutrophils.^84^ In addition, recent studies^66,85^ have demonstrated that microglia and neutrophils establish bidirectional communication in the inflammatory microenvironment of the CNS, mediated by cytokines, chemokines (CXCL1, CXCL8/IL-8, CCL2) and extracellular vesicles (EVs) released by both cell populations. These EVs carry microRNAs and regulatory proteins capable of modulating microglial polarization and inducing the release of NETs, forming an inflammatory feedback loop that aggravates neuronal damage. In experimental models of Alzheimer’s and Parkinson’s, this microglia–neutrophil interaction has been associated with the progression of neurodegeneration, synaptic dysfunction, and cognitive impairment, underscoring its importance as a therapeutic target (Figure 3).

**Figure 3:**
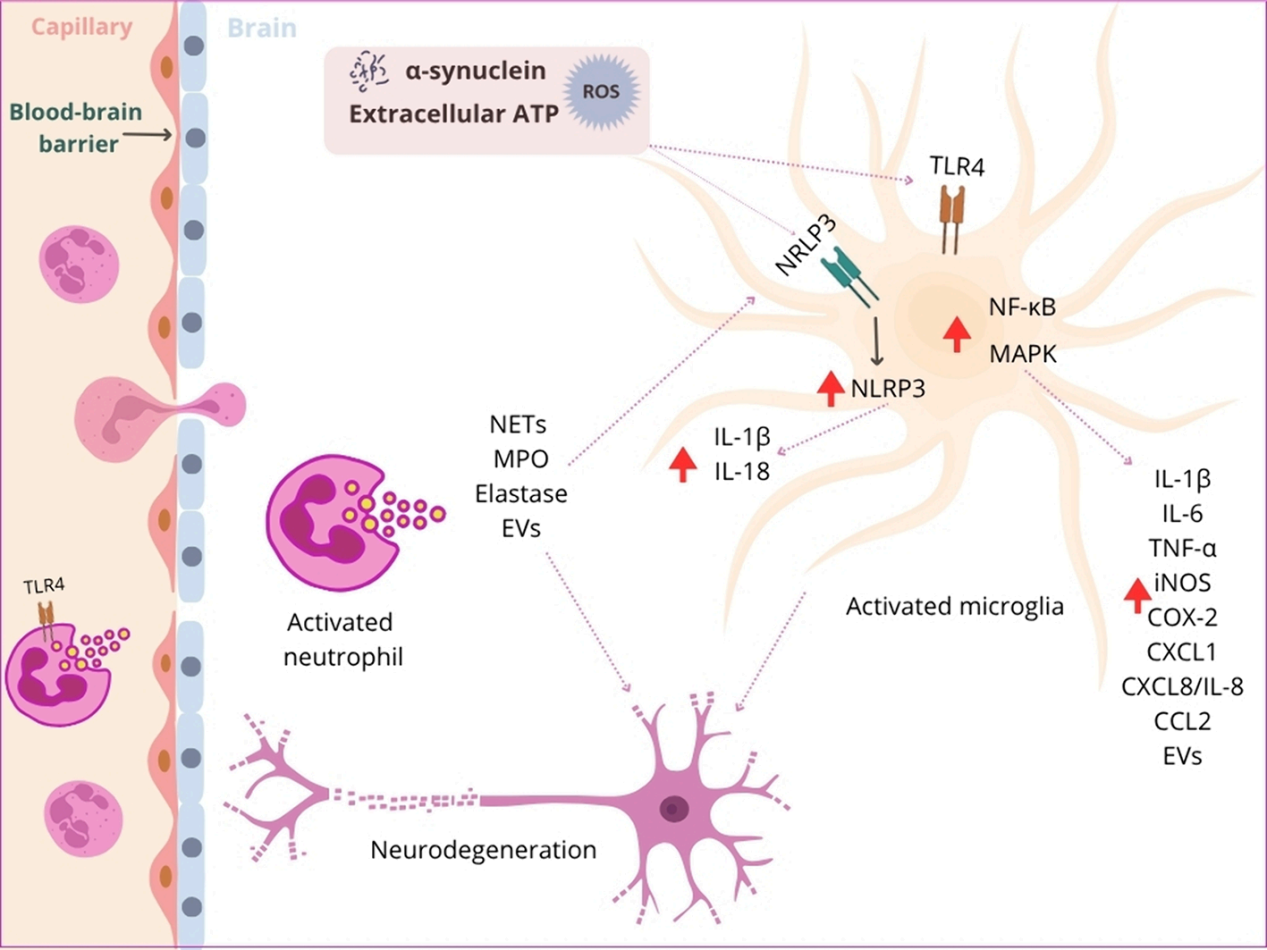
Neuroinflammatory pathways linking α-synuclein, microglial activation, and neutrophil-mediated neurodegeneration. Extracellular α-synuclein, ATP, and ROS act as DAMPs that cross the BBB and activate TLR4 and the NLRP3 inflammasome in microglia. The Subsequent activation of NF-κB and MAPK signaling pathways induces the expression of proinflammatory mediators, including IL-1β, IL-6, TNF-α, iNOS, COX-2, CXCL1, CXCL8/IL-8, CCL2, and EVs. In parallel, activated neutrophils release NETs, MPO, elastase, and EVs, amplifying microglial activation and promoting neuronal injury and neurodegeneration.

## 4 Central Nervous System and the COVID-19: Mechanistic Overlaps With Neurodegeneration

### 4.1 Alzheimer’s Disease

Alzheimer’s disease is the most frequent neurodegenerative disorder and the most common type of dementia, representing an important public health problem for the 21st century. It is estimated that more than 55 million people worldwide have Alzheimer’s or some other form of dementia. Alzheimer’s disease is characterized as a progressive neurological disease that affects approximately 5.3 million people aged 65 and over, and 200,000 young people (early onset Alzheimer’s) in the US. Therefore, as life expectancy increases, a greater number of people are affected by this disease, and it is estimated that by 2050, 16 million in the US alone will have Alzheimer’s disease and 135 million worldwide.^86^

Since the first descriptions of Alzheimer’s disease, a lot of progress has been made, and macroscopic and microscopic characteristics are now known. Macroscopically, Alzheimer’s disease is characterized by atrophy of the hippocampus and cerebral cortex, with primary involvement of the frontotemporal association cortex.^87,88^ Microscopically, protein aggregates of hyperphosphorylated tau protein are observed, which is physiologically involved in stabilizing microtubules in the neuronal cytoskeleton. Additionally, senile plaques are composed mostly of Aβ, produced by the cleavage of the amyloid precursor protein (APP).^42–90^

Different mechanisms are involved in the neuroinflammatory response in Alzheimer’s disease, and studies indicate different paths regarding the causality of the process. It has been proposed that inflammation arises in the presence of defective Aβ and tau proteins, leading to the onset or acceleration of disease progression. Others, however, argue that inflammation may be a defense mechanism against neurotoxicity in brain tissue affected by Alzheimer’s disease.^91^

The amyloid-β (Aβ) peptide-triggered chronic inflammatory process seems to have an important contribution to the neurodegenerative process in Alzheimer’s disease. The main inflammatory mediators observed in Alzheimer’s disease are produced by astrocytes and activated microglia surrounding the senile plaques.^92,93^ Prolonged and generalized activation of these glial cells in the brain correlates with the extent of brain atrophy and cognitive decline.^94,95^

The neuroinflammatory phenotype in Alzheimer’s disease is characterized by glial activation and a local acute inflammatory response mediated by cytokines, complement system activation, glutamate release, and induction of inflammatory enzymes, including iNOS and COX-2, with a consequent increase in the generation of NO, lipid mediators and ROS.^96^

Beta-amyloid and Tau are proteins recognized by microglial receptors. The interaction between these proteins and microglia activates inflammatory and immune pathways, resulting in the production of inflammatory mediators, such as cytokines and chemokines. This process also involves recognition by PRRs on microglia, such as TLRs and C-type lectin receptors (CLRs). This triggers the activation of microglia.^97^

When microglia are activated by pathological proteins such as beta-amyloid and Tau, they tend to adopt the M1 phenotype. This occurs because these pathological stimuli activate several inflammatory signaling pathways, such as the NF-κB pathway, MAPKs, and the TLRs signaling pathway. These pathways favor the expression of genes related to inflammation and the production of mediators such as reactive oxygen and nitrogen species that generate a neurotoxic environment, perpetuating microglial activation and disease progression.^94^

Pro-inflammatory cytokines (IL-1β, IL-6, and TNF-α) are involved in the initiation and amplification of the process, with the hippocampus being the CNS structure with the highest density of cytokine receptors, thus highlighting its vulnerability to the inflammatory process. Cytokines modulate complex mechanisms involved in various neural circuits, including those regulating thermoregulation, appetite, sleep patterns, and behavior. Evidence also suggests that cytokine networks are involved in the central mechanisms of memory and learning.^98,99^

Inflammation in the peripheral immune system also plays an important role in Alzheimer’s disease. In diseases such as Alzheimer’s disease, the BBB becomes more permeable, allowing peripheral immune cells and inflammatory mediators to migrate into the brain and interact with microglia, thereby amplifying the inflammatory response.^100^ The interaction between the peripheral and central immune systems in Alzheimer’s disease creates a vicious cycle of inflammation that contributes to neurodegeneration, so that peripheral inflammation can increase the permeability of the BBB and allow the entry of immune cells into the brain, activating microglia and producing an inflammatory response that further promotes the activation of peripheral immune cells, perpetuating inflammation. This inflammatory cycle contributes to neuronal death, synaptic dysfunction and the progression of Alzheimer’s disease, being one of the main mechanisms responsible for the cognitive impairment observed in the disease.^101^

### 4.2 Parkinson’s Disease

Parkinson’s disease is a neurodegenerative disorder characterized by resting tremor, postural instability, autonomic dysfunction, bradykinesia, and neuropsychiatric symptoms.^102^

Several biological hypotheses are proposed to explain the pathophysiology of Parkinson’s disease, including mitochondrial dysfunction, oxidative stress, protein aggregation, impaired autophagy, and neuroinflammation. Neuroinflammatory processes and abnormal immune response activation have been described as important players in the initiation and progression of the disease.^101,103–105^

Neuropathological hallmarks of Parkinson’s disease include: (i) deposition of misfolded protein aggregates in brain regions, including α-synuclein; (ii) progressive degeneration of dopaminergic neurons in the substantia nigra pars compacta (SNpc) with subsequent depletion of striatal dopamine, which is responsible for the classical motor symptoms.^106,107^ According to disease progression, nonmotor areas are also compromised by deposition of misfolded protein aggregates, leading to nonmotor symptoms such as sleep disturbance, cognitive impairment, olfactory dysfunction, and autonomic dysfunction.^108^

According to Gelders et al. (2018),^103^ inflammatory processes are directly involved in the etiology of Parkinson’s disease, especially the injury to the nigrostriatal pathway. Immune alterations in response to extracellular α-synuclein may play a critical role in Parkinson’s disease progression. Alpha-synuclein, can also interact with microglia through PRRs, such as TLRs. The interaction between these proteins and microglia activates the M1 pattern, resulting in the production of inflammatory mediators, inducing a local inflammatory response.^109,110^

Several genes encoding proteins associated with familial forms of Parkinson’s disease, including ATPase 13A2 (ATP13A2 gene), α-synuclein (PARK1 and PARK4), parkin (PARK2), and DJ-1 (PARK7), are involved in the regulation of microglial and astrocytic activation during neuroinflammation. Progressive dopaminergic loss observed in early Parkinson’s disease is associated with increased activation of microglial cells, expressing MHCII, ICAM1, and LFA1. In fact, these cells are observed in the substantia nigra of Parkinson’s disease patients.^111^ Aggregated α-synuclein is released from degenerating dopaminergic neurons, triggering a proinflammatory microglial phenotype characterized by overexpression of TNF-α, NO, IL-1β, and other proinflammatory microglial derivatives. These factors collectively modulate the neuroinflammatory process in Parkinson’s disease.^112,113^

Oxidative stress is a key factor in the complex degenerative cascade underlying dopaminergic neurodegeneration in all forms of Parkinson’s disease.^114,115^ Increased oxidative stress is found in the early stages of the disease, occurring before significant loss of dopaminergic neurons in the nigrostriatal region. Additionally, uncontrolled ROS generation can be seen as a potential factor causing dopaminergic neuron death, rather than being a secondary response to progressive neurodegeneration.^116^

The activation of microglia in the brain, in response to α-synuclein and other pathological factors, together with the infiltration of peripheral immune cells due to BBB dysfunction, contributes to chronic neuroinflammation and neurodegeneration. This interaction can be considered one of the main mechanisms responsible for the progression of Parkinson’s disease, as it generates a vicious cycle, in which inflammation in the brain attracts cells from the peripheral immune system, which, in turn, intensify inflammation in the CNS. This cycle perpetuates neurodegeneration where mitochondrial dysfunction, oxidative stress and continuous activation of microglia create a neurotoxic environment.^117,118^

### 4.3 COVID-19

COVID-19 is a pandemic caused by SARS-CoV-2 infection. This infection is associated with acute pneumonitis, fever, and cough. However, in a subgroup of patients, it can cause acute respiratory distress syndrome and severe pulmonary damage, hyperinflammation, and sepsis, which correlates with a poor prognosis for the disease. In the most severe cases, injuries to the cardiovascular system are observed, including in the kidneys, blood vessels, and heart. Vascular cells, especially endothelial cells, produce coagulation factors, leading to the formation of microthrombi and potential damage to circulatory system.^119^

The main mechanism of SARS-CoV-2 entry into the host cell involves the direct interaction of the spike S1 protein with Angiotensin-converting enzyme-2 (ACE2) in the host cell. There is also an important participation of cell proteases, including TMPRSS2 and ADAM17.^120,121^ Physiologically, ACE2 regulates the renin-angiotensin system by producing the anti-inflammatory angiotensin 1–7. The binding of SARS-CoV-2 reduces ACE2 expression,^122,123^ pushing the system toward a pro-inflammatory activation profile. This interaction causes increased production of cytokines (IFNs, IL-6, and TNF-α) and establishes, thereby influencing the inflammatory cascade observed in COVID-19. For detailed understanding of the mechanisms of SARS-CoV-2 internalization and replication in host cells, specialized reviews are recommended.^124–126^

In vitro studies have demonstrated that brain endothelial cells, pericytes, and astrocytes interact with SARS-CoV-2. Additionally, pericyte-like cells and brain organoids can be productively infected by SARS-CoV-2, leading to the production of type I IFN,^127^ a potent inflammatory cytokine involved in virus clearance. Notably, COVID-19 patients exhibit some neurological symptoms, including dizziness, anosmia, headache, and insomnia. The presence of SARS-CoV-2 in the brain has been observed in experimental models. Transgenic mice overexpressing human ACE2 display the presence of the virus in the brain.^128^ Experiments in nonhuman primates have demonstrated that the main route of CNS infection by SARS-CoV-2 is through the olfactory route.^129^ However, biopsies from autopsies from deceased COVID-19 patients displayed low or undetectable presence of viral RNA in the brain or cerebrospinal fluid, although it was also observed in ischemic areas, infarcts (ischemic and hemorrhagic), microglial activation, and T cell infiltration.^130,131^

Epidemiologic and experimental studies indicate that SARS-CoV-2 infection is associated with neuroinflammation and neurological disorders. A prospective study involving 60 hospitalized COVID-19 patients reported that during the acute phase of infection, 68% exhibited neurological symptoms such as mood alterations, fatigue, headache, myalgia, impaired mobility, and memory loss. During the follow-up phase, 55% of these patients presented evidence of neurological damage, predominantly affecting the central olfactory cortices and the white matter of the right hemisphere.^132^ Another follow-up study in COVID-19 survivors revealed that 19% of patients experienced cognitive decline, with signs of cognitive impairment observed after 6 months hospitalization.^133^ This was corroborated by the recent study Researching COVID to Enhance Recovery (RECOVER-Adult), which investigates the prevalence of long COVID in 13.647 individuals and observed that 63.8% and 65.8% of the infected people develop brain fog and dizziness, respectively.^134^ Apart from the significant impact on personal life, this condition can also have economic consequences, as these individuals are less likely to engage in full-time employment, leading to a reduction in workforce participation. This could cost approximately $170 billion in lost wages in the United States alone.^135^

Patients with neurodegenerative disorders such as Parkinson’s and Alzheimer’s disease may experience a decline in their clinical condition when infected with COVID-19.^136^ This worsening has a multifactorial cause and involves: I) the inflammation triggered by the virus, which can exacerbate existing symptoms and may even accelerate disease progression; II) patients with neurodegenerative diseases may have a compromised immune system, potentially reducing the capacity to combat viral infections effectively; III) presence of multiple comorbidities and frailties associated with neurodegenerative diseases, such as respiratory impairment, swallowing problems, physical frailty and malnutrition, which are aggravated by COVID-19 and IV) interruptions in the regular treatment of neurodegenerative diseases, such as administration of medications, physical and occupational therapies, which can lead to inadequate management of symptoms, increasing discomfort and clinical deterioration.^137,138^

Parkinsonism has also been observed as a consequence of encephalopathies caused by viral infections, and it has been suggested that COVID-19 may affect dopaminergic neurons. One possible mechanism involves the interaction of SARS-CoV-2 and α-synuclein, leading to the formation of amyloid fibrils, a pathological hallmark observed in Alzheimer’s disease and Parkinson’s disease.^139–141^ In addition, the N-terminal APP, the precursor of Aβ, interacts with the Spike protein of SARS-CoV-2, facilitating the viral entry and exacerbating symptoms of Alzheimer’s disease in animal models.^142^ Of note, increased expression of oligoadenylate synthetase 1 (OAS1)—a gene associated with Alzheimer’s disease risk—has been observed in the microglia of COVID-19 patients as well. OAS1 is coexpressed with interferon-responsive genes, which are typically elevated during viral infections. Significant association between COVID-19 and neuroinflammation is also related to cerebral microvascular injury, a process implicated in both Alzheimer’s disease and vascular dementia. This may be partially explained by studies which state that individuals with the Alzheimer’s disease risk allele (APOE E4/E4) presented reduced levels of antiviral defense genes compared to the APOE E3/E3 genotype.^143^

#### 4.3.1 Neuroinflammatory Mechanisms Involved in COVID-19

Mechanisms related to the inflammatory response in the brain are still under debate. Infected patients exhibit low levels of viral particles in the brain. Although SARS-CoV-2 interacts with receptors in brain cells and activates inflammatory response, it does not replicate efficiently, similar to what has been observed in cells from the vasculature.^135,144^ Therefore, it is most likely that the possible damage might be secondary to systemic inflammation, with synergistic contribution from hypoxia/ischemia and oxidative stress. Notably, post-mortem brain tissue from COVID-19 patients has shown expression of ORF3a, an accessory protein of SARS-CoV-2 that is involved in the activation of SUR1 ion channels, enhanced intracellular ion influx, NF-κB activation, and subsequent inflammatory responses in astrocytes.^145^ In addition, long-COVID-19 patients exhibit reduced plasma levels of the antioxidant GSH.^146^

Studies using experimental models expressing human ACE2 have demonstrated that SARS-CoV-2 infection induces disruption of the BBB and enhances innate immune activation,^147^ with an increased proportion of macrophages in the brains. These infiltrating cells produce pro-inflammatory cytokines amplifying the neuroinflammatory response.^148^ Similar findings have been reported in post-mortem brain samples from COVID-19 patients.^149,150^ Of note, data from the UK Biobank demonstrated a reduction in gray matter thickness and a reduction in global brain size after COVID-19 infection.^151^ Whether this deleterious effect can be partially reversed or persists in the long term remains to be determined. Experimental models demonstrated the presence of SARS-CoV-2 in SNpc, which was correlated with increased levels of IL-1β, Bradykinin B1 receptor, and ADAM17.^152^ However, it is still elusive whether these mechanisms affect the dopamine neurons in the SNpc and contribute to Parkinson’s disease.

Spatial analysis by imaging mass cytometry revealed that brains from COVID-19 patients exhibit clusters of CD4+ and CD8+ lymphocytes, B cells, and macrophages. The presence of perforin and granzyme B indicates activation of CD8^+^ T cells, suggesting cytotoxic activity and potential brain tissue injury.^153^ In addition, multiOMICs studies demonstrated that brain tissues from deceased individuals exhibit type I IFN (IFNα and IFNβ), followed by increased expression of signal transducer and activator of transcription 1 (STAT1), an inflammatory transcription factor involved in IFN inflammatory pathways.^154^ In addition, an autopsy platform study in COVID-19 patients demonstrated that inflammatory response in peripheral organs is linked to increased IL-1 and IL-6 specific virus-sensing PRR fingerprints in different areas of the brain, which are associated with mitochondrial failure and cell death occurring at sites of viral antigen-associated vascular inflammation, marking areas of synapse and myelin injury.^155^ Moreover, proteomic analysis of the cerebrospinal fluid (CSF) from these patients demonstrated various protein biomarkers also found in CSF from patients with cognitive impairment and early onset Alzheimer’s disease, including CASP-8, IL-18, and CSF-1.^155^

SARS-CoV-2 also activates innate immune receptors, including TLR2 and TLR4, and induces the production of pro-inflammatory cytokines (e.g., IL-6, TNF-α, IFN-γ, IL-18, and IL-1β) and ROS production regardless of virus internalization.^156–158^ This can be involved in cell injury, as it was demonstrated that IFN-γ and TNF-α can synergistically induce tissue injury by inducing PANoptosis, which is a specific type of cell death mechanistically different from apoptosis, pyroptosis, and necroptosis, since it is independent of activation of caspase-3, gasdermin, or RIPK3 pathways, respectively.^159^

The NLR family NLRP3 inflammasome complex is an important component of the innate immune response expressed in various cells, including microglia. It has also been implicated in various inflammatory diseases, including COVID-19.^160^ In addition, colocalization of SARS-CoV-2, ACE2, and NLRP3 within neurons, astrocytes, and microglia was observed in brain biopsies from COVID-19 patients.^161^ The NLRP3 inflammasome comprises the NLRP3 protein, the adaptor molecule apoptosis-associated speck-like protein containing a CARD (ASC), and caspase-1. Upon activation, this complex promotes the maturation and release of the pro-inflammatory cytokines IL-1β and IL-18. NLRP3 activation is triggered by PAMPS, which are conserved motifs associated with pathogens, or by DAMPS, endogenous molecules released during cell injury. These include cell-derived nucleic acids, fatty acids, heat shock proteins, and HMGB1 (high-mobility group box-1).^162^

Stimulation of microglia with the S1 subunit of the SARS-CoV-2 spike protein has been shown to increase gene expression of NLRP3 and production of IL-1β, IL-6, and TNFα.^163^ This activation seems to be dependent on the interaction between the virus and ACE2.^160^ Intracerebral injection of S1 protein in experimental models resulted in cognitive impairment, neuronal loss, and neuroinflammation, effects that were significantly reduced in global NLRP3-deficient animals and microglia NLRP3-deficient cells.^163^ In addition, the *NLRP3* inflammasome genetic variants are associated with disease severity in COVID-19 patients, especially in elderly male individuals.^164^

The inflammatory response in the brain is the main link between COVID-19 with neurodegenerative disorders, including Alzheimer’s disease and Parkinson’s disease (Table 1). Although there is a lack of large-scale data demonstrating causative effects of COVID-19 on Parkinson’s disease or Alzheimer’s disease, molecular studies indicate similar inflammatory pathways. In addition, deficient gliovascular communication, microglial aberrant activation,^155^ reduced endothelial nitric oxide synthase (eNOS) and vascular dysfunction^165^ observed in COVID-19 patients and experimental models may contribute, accelerate, or exacerbate neurodegenerative processes. Moreover, patients with long COVID syndromes exhibit increased signal of 18-kDa translocator protein (TSPO) on positron emission tomography, suggesting microglial reactivity, which has been associated with depressive symptoms and cognitive dysfunction.^166^ Whether this inflammatory phenotype disappears or persists over time is still under investigation. Importantly, epidemiological data from the Chinese Longitudinal Healthy Longevity Survey (CLHLS) patients demonstrated an increase in cognitive impairment prevalence from 4.3% in pre-COVID-19 to 6.8%, post-COVID-19 in older adults.^167^

**Table 1:** Molecular Mechanisms Involved in Alzheimer’s Disease, Parkinson’s Disease, and COVID-19^a^

**category**	**Alzheimer’s disease**	**Parkinson’s disease**	**COVID-19**
immune mediated response (innate)	microglia; astrocytes; NLRP3 inflammasome; TLR2/4 activation	activated microglia, astrocytes; NLRP3 inflammasome; TLR2/4 activation	activated microglia, astrocytes, macrophage accumulation, NF-κB, NLRP3
immune mediated response (acquired)	CD16/32, CD86, CD40	CD16/32, CD86, CD40, MHCII, ICAM1, and LFA1	activated CD4 and CD8 T cells
cytokines/chemokines	IL-2; IL-8; CCL2; CCL3/4 and CCL5; IL-1β; IL-6; TNF-α and COX-2	CXCL1, CXCL8/IL-8, CCL2, TNF-α, IL-1β	IL-1, IL-6 IFN, STAT1, TNF-α, IL-1β, IL-18
oxidative stress	ROS; NO; MDA; OH·and O_2_^.‑^	ROS; NO; MDA; hydroxyl radical and O_2_^.‑^	ROS, reduced GSH
clinical markers	uric acid; hyperphosphorylated tau protein; protein *A*β (amyloid plaques and neurofibrillary tangles)	α-synuclein; uric acid; and depletion of striatal dopamine	α-synuclein; tau protein
clinical manifestations	cognitive impairment; memory loss	Resting tremor; postural instability; autonomic dysfunction; bradykinesia, and neuropsychiatric symptoms	long-COVID-19 syndrome, including fatigue and brain fog

a Legend: Coronavirus disease 2019 (COVID-19); Cluster of Differentiation 16/32 (CD16/32); Cluster of Differentiation 86 (CD86); Cluster of Differentiation 40 (CD40); NOD-, LRR- and pyrin domain-containing protein 3 (NLRP3); Toll-like receptor 2/4 (TLR2/4); Nuclear factor kappa B (NF-κB); Interleukin-2 (IL-2); Interleukin-8 (IL-8); Interleukin-1 beta (IL-1β); C–C motif chemokine ligand 2 (CCL2); C–C motif chemokine ligand 3/4 (CCL3/4) and C–C motif chemokine ligand 5 (CCL5); Interleukin-1 beta (IL-1β); Interleukin-6 (IL-6); Tumor necrosis factor alpha (TNF-α); Cyclooxygenase-2 (COX-2); Reactive oxygen species (ROS); Nitric oxide (NO); Malondialdehyde (MDA); Superoxide anion (O2.-); Hydroxyl radical (OH·); and Glutathione (GSH).

## 5 Therapeutic Implications and Future Directions

The importance of neuroinflammation has been observed in patients with neurodegenerative disorders and supported by convincing experimental data.^168,169^ It is reasonable to hypothesize that reducing ROS bioavailability through antioxidants, ROS scavengers, and Nox inhibitors or the anti-inflammatory therapy would have major beneficial effects. Although animal models have indicated beneficial effects of treatment with antioxidants, including apocynin, tempol, NXP031 (a combination of aptamin C and vitamin C), and antioxidant-rich diets, there is still a lack of data from robust clinical trials.^170–172^ Similar associations are observed in the inflammatory response. While the role of inflammasome activation has been demonstrated in the pathophysiology of Alzheimer’s disease and Parkinson’s disease,^173^ its clinical evidence remains elusive. Nevertheless, oxidants and inflammatory mediators are considered important clinical markers for neurodegenerative diseases. For instance, the EXSCEL randomized placebo-controlled trial demonstrated that exenatide, a glucagon-like peptide receptor agonist, reduced inflammatory proteins associated with Alzheimer’s disease, which correlated with decreased levels of serum inflammatory markers ficolin-2, plasminogen activator inhibitor 1 (PAI-1), and soluble vascular cell adhesion protein 1 (sVCfAM1).^174^ Corroborating this data, a clinical study showed that the administration of exenatide reduced motor symptoms of Parkinson’s disease.^175^

Agents used to treat comorbid conditions associated with increased risk of Alzheimer’s disease, such as metabolic and vascular disorders, are promising therapeutic candidates. Sodium–glucose cotransporter-2 inhibitors (SGLT2i), widely used for type 2 diabetes (T2D) and cardiovascular disease, have emerged as promising candidates. Supporting these observations, a clinical study using the SGLT2i Empagliflozin intranasal demonstrated reduced tau-related processes and reduced inflammatory biomarkers, including IL-2, IL-7, and neurogranin.^176^

A preliminary clinical study with Parkinson’s disease patients evaluated the immunomodulatory effect of sargramostin, a recombinant granulocyte-macrophage colony-stimulating factor (GM-CSF) administered subcutaneously for 56 days. The results suggested a potential therapeutic gain by modulation of immune response, but it was also registered as adverse events.^177^

The reasons for such disappointing or inconclusive clinical results remain unclear and can be multifactorial. It is possible that we still have not identified the optimal treatment route as well as the most effective target on the oxidative stress signaling pathways. Additionally, factors such as the choice and dosage of drugs, treatment duration, patient selection criteria, and the lipid solubility properties of the antioxidants used may also play significant roles in the observed variability of therapeutic effects. Moreover, intellectual property rights might limit global access to innovative neuroprotective therapies, as patent costs, regulatory barriers, and inequities can hinder their clinical translation.^178^ In addition, the ethnic background must be considered in clinical trials, such as immune-based therapies.

Considering Parkinson’s disease and Alzheimer’s disease, evidence supports the need to develop tools that allow early diagnosis of these diseases. As studies have suggested that the immune response appears to play an important role in the onset and progression of these diseases, analyzing the expression or concentration of markers associated with the activation of neutrophils and microglial cells early, before motor and cognitive symptoms, may be strategic. An early diagnosis could potentially support the effective use of anti-inflammatory, antioxidant, and immunomodulatory drugs, which might slow or even help control the progression of the diseases.

Finally, we highlight the advantages of new therapeutic approaches, such as the use of nanoparticles, which have been gaining prominence in research into neurodegenerative diseases, especially in Parkinson’s disease. Furthermore, biomaterials, including nanoparticles, hydrogels, and scaffolds, have emerged as promising neuroprotective agents due to their biocompatibility, biodegradability, targeted delivery, and controlled release, enhancing the therapeutic potential in CNS disorders.^179^ One of the main advantages we can mention is the ability to cross the BBB, one of the biggest obstacles to the effectiveness of therapeutic substances. Recent studies have shown that nanoparticles of silver, gold and other formulations effectively prevented progressive neurodegeneration in Parkinson’s disease. Therefore, these advances open up new possibilities for the development of more effective, and targeted treatments, capable of acting directly on the affected areas of the CNS.^180,181^

## 6 Conclusion

It is now increasingly recognized that the interaction of oxidants and immune system activation plays an important role in the neuroinflammatory response associated with Parkinson’s disease and Alzheimer’s disease. Emerging evidence also suggests a potential involvement of COVID-19 in CNS inflammation, although further long-term cohort studies and experimental models are required to validate these associations. This response occurs through interactions between the central and peripheral immune systems, with microglia and neutrophils contributing to neuronal dysfunction and death. The precise molecular pathways of these interactions remain elusive. Therefore, a better understanding of these mechanisms will help define how these immune cells protect or induce neurotoxicity, and at which stage of the disease these events occur. Such insights will allow a better understanding of these disorders and the identification of potential therapeutic targets that could improve current pharmacotherapy.
